# Deep temporal networks for EEG-based motor imagery recognition

**DOI:** 10.1038/s41598-023-41653-w

**Published:** 2023-11-01

**Authors:** Neha Sharma, Avinash Upadhyay, Manoj Sharma, Amit Singhal

**Affiliations:** 1 Department of Electronics and Communication Engineering, Bennett University, Greater Noida, 201310 India; 2 Department of Electronics and Communication Engineering, NSUT, New Delhi, 110078 India

**Keywords:** Computational biology and bioinformatics, Medical research

## Abstract

The electroencephalogram (EEG) based motor imagery (MI) signal classification, also known as motion recognition, is a highly popular area of research due to its applications in robotics, gaming, and medical fields. However, the problem is ill-posed as these signals are non-stationary and noisy. Recently, a lot of efforts have been made to improve MI signal classification using a combination of signal decomposition and machine learning techniques but they fail to perform adequately on large multi-class datasets. Previously, researchers have implemented long short-term memory (LSTM), which is capable of learning the time-series information, on the MI-EEG dataset for motion recognition. However, it can not model very long-term dependencies present in the motion recognition data. With the advent of transformer networks in natural language processing (NLP), the long-term dependency issue has been widely addressed. Motivated by the success of transformer algorithms, in this article, we propose a transformer-based deep learning neural network architecture that performs motion recognition on the raw BCI competition III IVa and IV 2a datasets. The validation results show that the proposed method achieves superior performance than the existing state-of-the-art methods. The proposed method produces classification accuracy of 99.7% and 84% on the binary class and the multi-class datasets, respectively. Further, the performance of the proposed transformer-based model is also compared with LSTM.

## Introduction

Electroencephalogram (EEG) has been the most intriguing technique in the medical field since its inception in 1929 by Hans Berger due to its non-invasive nature and the vast range of applications in neuroscience^1^. One of the applications of EEG signals is brain–computer interface (BCI) systems which provide a direct channel of interaction between the external equipment and the brain’s electrical activity. The important paradigm of EEG-based BCI systems is motor imagery (MI) based systems. MI signals are the brain signals which are recorded while a person imagines the movement of a body organ. These signals are transferred to external environments such as robots, exoskeletons, and autonomous vehicles^2^. MI-EEG-based BCI applications in the fields of medicine, robotics, and gaming have garnered a great deal of interest from researchers over the past two decades^3^. Motion recognition using EEG data, primarily MI data, has the potential to address the plight of locked-in or paralysed persons and improve their quality of life^4^.

Prior to the development of deep learning techniques, researchers used conventional signal processing techniques to analyse MI EEG signals. In a conventional pipeline, raw EEG data is pre-processed using dimensionality reduction by independent component analysis (ICA)^5^, multiscale principle component analysis (MSPCA)^6^ and denoising techniques such as regression or blind source separation (BSS)^7^ may be applied. After pre-processing, data may be decomposed using short-time Fourier transform (STFT)^8^, wavelet decomposition (WD)^9^, empirical mode decomposition (EMD)^6,10^ or Fourier decomposition method (FDM)^11,12^. The statistical and temporal features^13^ are extracted from the decomposed signal and classified using several machine learning classifiers, such as support vector machine(SVM), k-nearest neighbour (kNN), Naïve Bayes (NB), and decision tree (DT)^14,15^. The conventional pipeline may not perform well for multi-class scenarios in large datasets. With the advent of deep learning techniques, researchers have explored their usage in MI-EEG signal classification. Various deep learning approaches based on convolutional neural network (CNN)^16^, recurrent neural network (RNN)^17^, and long-short-term memory (LSTM)^10^ have been investigated. Taheri et al.^18^ selected the most discriminant features using a CNN by making a triple frame matrix, combining EMD, Fourier transform, and common spatial patterns. Li et al.^16^ used a CNN with an LSTM to extract features by connecting them in parallel. Thereafter, classification is performed using a fully connected layer on combined features.

In contrast to CNN-based models, LSTM has shown remarkable improvement in the classification of time-series MI raw data^19^. The network is capable of modeling the short-range time-sequence information, however, its performance is limited because of the gradient diminishing issues in long sequence data and computationally expensive serial processing of data. The transformer has emerged as a good replacement for LSTM as it works well with long time-series data and can process the data in parallel. The transformer uses a self-attention mechanism to deferentially weigh every part of the input which provides efficient features for better recognition. Some recent works^20,21^ proposed spatial, temporal, and spectral feature extraction on pre-processed data. However, these features are not an exact representation of the complete information as some information may get lost in the pre-processing step. Hence, in this paper, we have customized the conventional transformer and LSTM models for the recognition of motor movements. These frameworks have been adapted for raw EEG MI data recognition. To the best of our knowledge, no previous studies have explored the effectiveness of transformers on raw MI data. Existing research in MI recognition has typically involved preprocessing the data before feeding it into a model, resulting in additional computational complexity, increased latency time, and hardware requirements. In contrast, our methodology utilizes raw EEG data as input, which is advantageous for real-time applications, as EEG data is not in image form.

The key contributions of this work are:

- Proposed a deep temporal network-based model for motor recognition on unprocessed (raw) MI EEG signal.
- Performance comparison of LSTM and transformer in binary and multi-class scenarios.
- Comparison of the proposed networks with existing state-of-the-art methods (with or without pre-processing steps) on raw MI EEG data.

The remaining paper is structured as follows: A brief survey of the existing schemes is presented in the related work section. The methodology section provides details about the network architecture, dataset preparation, training and testing, as proposed in this work. Later on, results and discussion are presented for binary and multi-class recognition. Finally, we conclude the article by discussing the outcomes of this study and outlining future directions.

## Related work

In the last decade, conventional signal processing techniques have been used extensively to analyse MI signals. Techniques based on the time domain, frequency domain, and time-frequency domain are employed to extract features in^22^. STFT^22^ can be employed for signal decomposition on multi-class datasets. This technique is quite simple, but it provides a confusing overlap boundary in data decomposition. For a narrow window, it provides poor frequency resolution, while a wider window results in poor time resolution. Wavelet-based methods can resolve this problem. Ganorkar et al.^23^ used WD along with SVM classifier. They used different wavelets and kernels to attain a good accuracy on multiclass dataset. However, there is a problem of oscillations at signal discontinuities. Taran et al.^24^ proposed the analytic intrinsic mode functions (IMF) based features for classification. EMD and Hilbert transform (HT) collectively generate IMFs, which are passed to a least squares SVM (LS-SVM) classifier. Due to the mode mixing issue with the EMD approach, IMF functions cannot be accurately approximated. Bhattacharyya et al.^25^ suggested the use of Fourier–Bessel series expansion (FBSE) to improve empirical wavelet transform (EWT). Signals are segregated into different narrow-band components using wavelet-based filter banks, and then the normalized HT is employed to evaluate the amplitude envelope and instantaneous frequency functions. Discrete wavelet transform (DWT) is considered to decompose the signal into narrow-band signals that are further decomposed using EMD^26^. From the signal components thus obtained, approximative entropy is computed for classification using the SVM technique. One of the limitations of EMD is the lack of a proper mathematical background. Kumar et al.^27^ used the FDM method for the detection of seizure and non-seizure events from EEG signals. However, the selection of the number of sub-band components becomes an important step in FDM technique. The features extracted from all these techniques are hand-crafted features. For larger datasets, these strategies may be less appropriate as compared to the self-learning methods.

Since the deep learning techniques require a large amount of data for adequate training, they perform well with larger datasets. The time-domain MI data can also be converted into time-frequency representation for feature extraction and classification as an alternative to conventional classification techniques considering time-domain data directly. This strategy can provide us positive outcomes on pre-trained networks. Kumar et al.^28^ suggested spatial filtering using common spatial filtering (CSP) and performed experiment with 10 cross fold validation. Zhou et al.^29^ offered an innovative technique based on wavelet envelope analysis and LSTM classifier. Luo et al.^30^ outlined a method in which they produced spatial features, which were then fed to a RNN for classification using cropped time slices of the signals. To combat memory distractions, the RNN design was modified to incorporate the widely used gated recurrent unit (GRU) and LSTM unit. Kumar et al.^31^ suggested a model with CSP and LSTM network. The authors have also performed a t-test and obtained p-values. Miah et al.^32^ proposed a technique for high dimensionality and dynamic behaviour of EEG signals. The authors suggested a novel technique CluSme based on clustering-based ensemble technique for this challenge.

Further, Khademi et al.^33^ analyzed a CNN along with an LSTM classifier. They converted 1-D data into images for a multiclass dataset. Tiwari et al.^34^ suggested a deep neural network for recognition of motion. Authors have used spectral features for binary class classification. Song et al.^20^ performed spatial filtering on the dataset and then performed an attention mechanism on the dataset. But they used a subject-specific model. On the other hand, a recent work by Ma et al.^21^ used an attention mechanism for MI classification. They used both temporal and spatial information for a multiclass dataset. Kostas et al.^35^ down-sampled the EEG data and then mapped the data using a transformer encoder. However, the spatial and temporal information collapsed in this model. Further, Tao et al.^36^ considered a gated transformer. Xie et al.^37^ proposed numerous models based on the input provided in the transformer module such as spatial input, temporal input, and different CNN models output as input to the transformer. But the accuracy attained by above mentioned model is still lower than ours after increasing so much complexity . Moreover, the pre-processing step adds computational overhead to the overall model.

## Methodology

This section describes the proposed architectures. The MI EEG signal analysis is performed using LSTM and temporal transformer architecture. Both these networks are trained on the same dataset and in similar settings. We implemented both models to compare their performance on long MI EEG signal sequences.

### LSTM-based architecture

**Figure 1:**
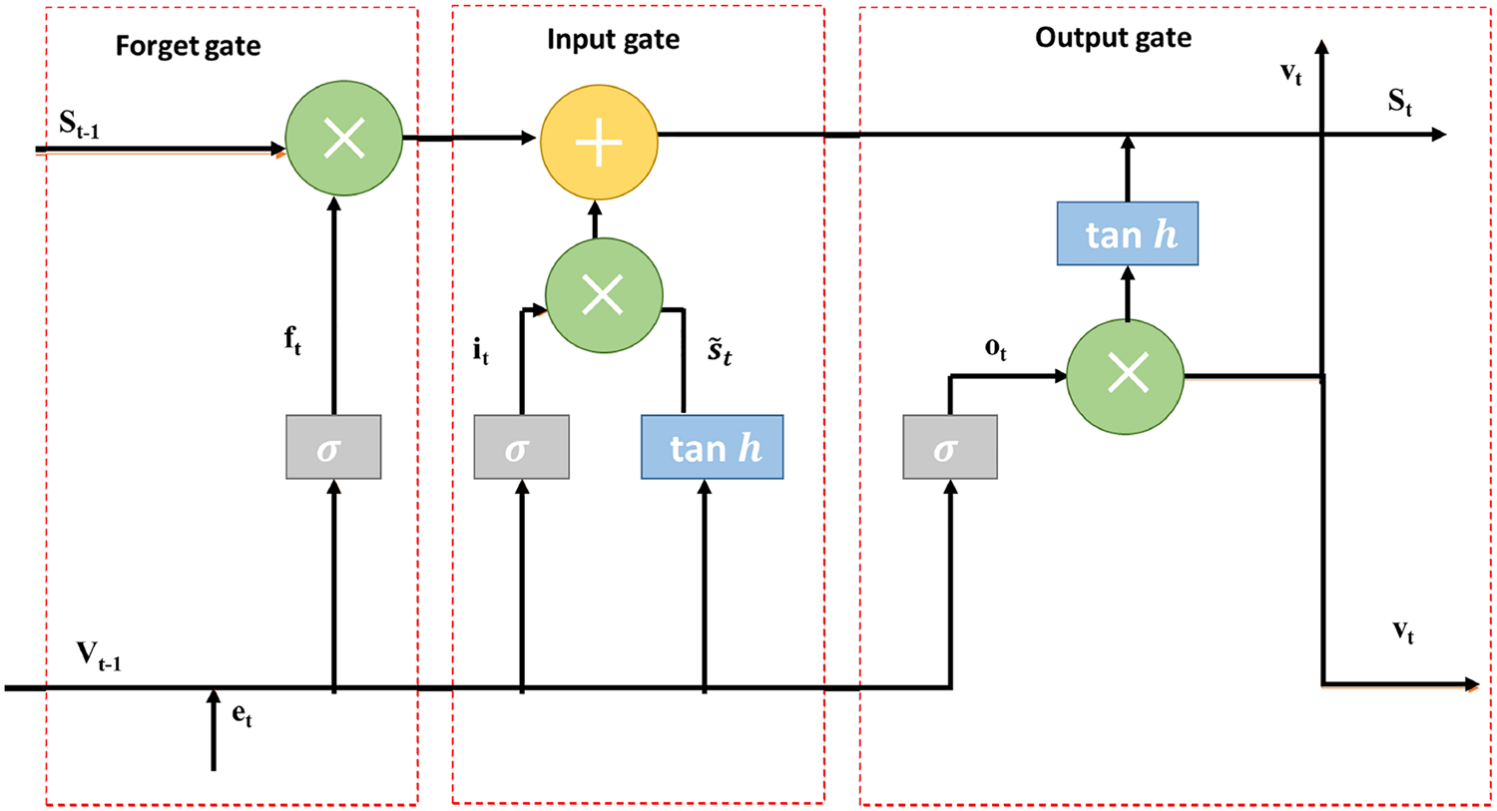
Architecture of LSTM block.

This architecture uses a fully connected layer with input sequence $X \in {\mathbb {R}}^{T_{s} \times N_{e}}$, where $N_{e}$ is the number of electrodes used for collection of MI-EEG signals and $T_{s}$ ($T_{s} < N_{d}$) is the time sequence, as the input layer. The total length of time series data is denoted by $N_{d}$. The details regarding the dataset preparation are provided later in the Experiments section. These neurons are passed to an LSTM Network with 100 hidden units. The LSTM is used for processing the time-series data and extracting temporal features. A typical diagram of LSTM architecture^29^ is depicted in Fig. 1. It consists of different memory blocks called cells, where *e* represents the input signal, *b* denotes the bias value, $f_t$ refers to the activation vector of forget gate, *w* is the weight matrix associated with the signal, $v_{t-1}$ is the output of the prior cell, *s* is the cell state vector, $\widetilde{s_t}$ and $i_t$ denote the activation vectors of cell state and input, respectively, $v_t$ is the output from the output gate^19^.

**Figure 2:**
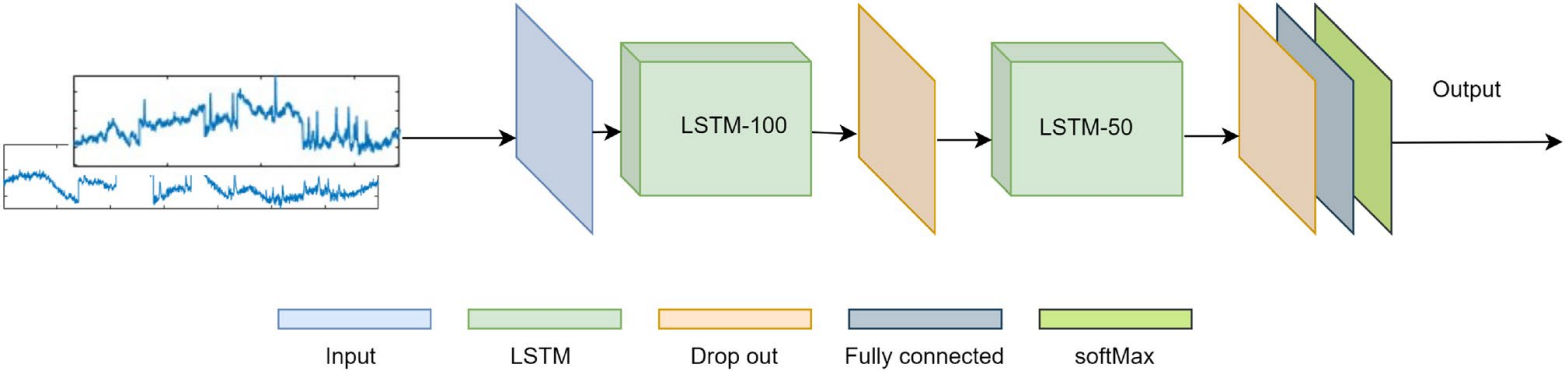
Proposed architecture for LSTM-based MI EEG signal recognition.

The LSTM-100 (LSTM with 100 hidden units) has a dropout of 0.2 to regularize the learning and prevent over-training. The output of this dropout layer is fed to another LSTM with 50 hidden units (LSTM-50). The ReLU activation function is used for each node. LSTM-50 also has a dropout of 0.2. This layer is connected with another neural network layer with $O_{N_{c}}$ neurons for each class. To obtain the probability vector for each class, the output of these neurons is passed to the Softmax layer. The proposed architecture is shown in Fig. 2.

### Transformer

**Figure 3:**
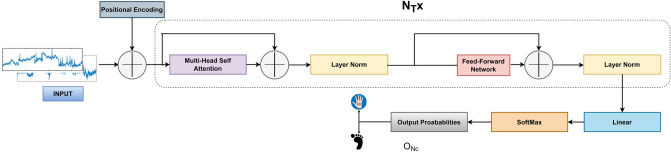
Proposed architecture for transformer-based MI EEG signal recognition.

The architecture for transformer is shown in Fig. 3. The sequential data is encoded at each time stamp provided by $N_{e}$ electrodes. The input sequence $X \in {\mathbb {R}}^{T_{s} \times N_{e}}$ is embedded before passing to the attention layers. Each $x_i \in {\mathbb {R}}^{1\times N_e}$, an element of *X*, is a token which is converted to the vector using the embedding layer. The positional embedding $P_{Pos\_embedd} \in {\mathbb {R}}^{T_{s} \times d_{model}}$ of input data is obtained using a linear layer. The embedded vectors are further positional encoded to form the embedded input sequence matrix. These matrices consist of spaces where similar vectors are close to each other and are called embedding spaces, which perform the function of mapping the data. Figure 3 represents the encoder part of the transformer which comprises several layers of the same structure. Every encoder layer includes multi-head attention and a feed-forward layer along with a hidden layer. Both layers are followed by a normalization layer and the remnant is also fed to both of the layers. Four transformer blocks are used in the proposed architecture. The output of the last transformer encoder block is fed to a neural layer $O_{N_{c}}$ which has one neuron for $N_{c}$ class. This neural layer is accompanied by the Softmax layer that gives the probability vector for each class.

Various layers of the transformer module are explained below.

#### Scaled Dot-product attention

**Figure 4:**
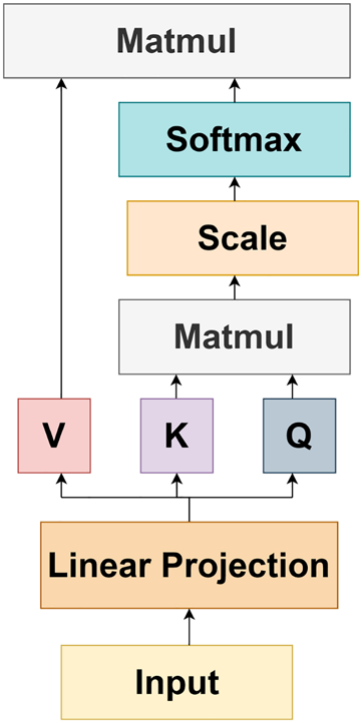
Scaled-Dot product attention.

The architecture of scaled dot-product is illustrated in Fig. 4. Input data is mapped to three kinds of information sources: query (*Q*), key (*K*) and value (*V*), whose dimensions are $d_q$, $d_k$ and $d_v$, respectively. For obtaining the weights, we calculate the dot product of the query with all the keys, scale it by $1/\sqrt{d}_k$ and then apply the SoftMax function. The scale factor $\sqrt{d}_k$ helps to generate a smoother SoftMax result. This computation is performed on all the channels. Therefore, the output matrix is computed as:

1 $$\begin{aligned} Attention\left( Q,K,V\right) =SoftMax \left( \frac{QK^T}{\sqrt{d}_k} \right) V. \end{aligned}$$

The two most prevalent attention mechanisms are additive and dot product^38^. Dot product attention is much faster and space efficient. For a large value of $\sqrt{d}_k$, dot product attention grows large in magnitude due to which SoftMax function goes into a region, where it exhibits an extremely small gradient. Hence, we have scaled the dot product by $1/\sqrt{d}_k$.

**Figure 5:**
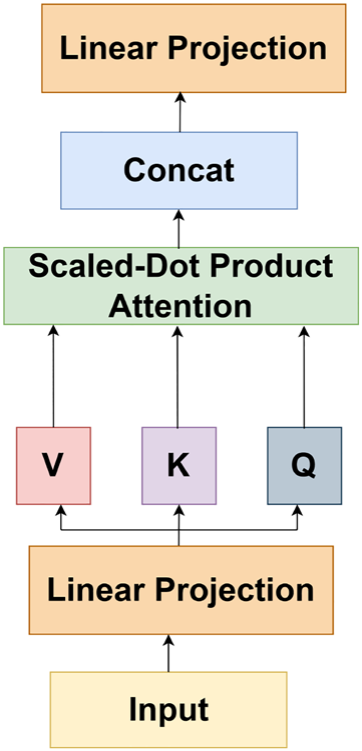
Multi-head attention.

#### Multi-head attention

The schematic of the multi-head attention mechanism constituting the scaled dot-product attention module is shown in Fig. 5. To obtain a set of *Q*, *K*, and *V* with dimensions $d_q, d_k$, and $d_v$, the features are linearly projected. One head is a scaled dot-production module of *Q*, *K*, and *V*, and similarly, a total of *h* heads are there for all the values of *Q*, *K*, and *V*. These outputs are concatenated and fed to a linear layer to produce the final output.

2 $$\begin{aligned} Multihead (Q,K,V) = Concat (head_0 \ldots head_h) W_O, \end{aligned}$$

where $head_i = Attention (QW_i^Q,KW_i^K,VW_i^V)$ and projections $(W_O)$ are parameter matrices. In this work, we consider $d_q = d_k = d_v = d_{model}/ h =256$. Due to parallel computing, the computational cost is the same as single-head attention, and the efficiency gets boosted.

#### Position encoding

To incorporate positional information into the Transformer model, we apply positional encoding to the output of the Embedding layer. The positional encoding ensures that the model understands the relative positions of tokens in the input sequence. The sine and cosine functions^39^ of different frequencies used in this work are :

3 $$\begin{aligned} PE_{\left( Pos,2i\right) }=sin{\left( Pos/{10000}^{2i/d_{model}}\right) } \qquad \qquad \qquad \qquad PE_{\left( Pos,2i+1\right) }=cos\left( Pos/{10000}^{2i/d_{model}}\right) . \end{aligned}$$

For positional encoding, *pos* represents the position and *i* denotes the dimension. The output of the position embedding is $X \in {\mathbb {R}}^{T_{s} \times d_{model}}$ which is same as $P_{pos\_embedd}$. Thereafter, the positional encoding matrix is added element-wise to the output of the Embedding layer, resulting in the final input to the Transformer model.

#### Feedforward network

The feed-forward network is a sub-layer of encoder layers. This comprises two fully connected layers. The output of multi-head attention and residual input is also fed to the feed-forward network. This function is expressed as:

4 $$\begin{aligned} FFN\left( X^\prime \right) =Relu{\left( X^\prime w_i^1+b_i^1\right) w_i^2}+b_i^2, \quad X^\prime =X+ Multihead (X). \end{aligned}$$

The last feed-forward layer before the final linear layer have output dimension of 1024.

The transformer network excels in handling time sequence data, such as the continuous data produced by electrodes capturing the activity of different brain regions during an event. This time sequence data is strongly correlated, reflecting the interconnectedness of brain activity over time. The transformer encodes this data by leveraging its learnable attention mechanism, which captures the dependencies and correlations between the electrode outputs at different time steps. By attending to relevant information, the transformer effectively models the temporal relationships within the sequence, allowing it to extract meaningful features through its multi-layer perceptron (MLP) layer. These features are specifically designed to maximize the correct prediction of the event, as the network iteratively performs this encoding and feature extraction process. The final output of the transformer, after passing through a Softmax layer, represents a probability distribution over different event classes based on the encoded features and the learned correlations within the time sequence data.

### Training loss

We have used categorical cross-entropy loss to train both LSTM and transformer models. The sparse categorical cross-entropy loss is a commonly used loss function in classification problems. It is defined as:

5 $$\begin{aligned} L_E=-\sum _{k=1}^{n}{t_klog{\left( P_k\right) }}, \end{aligned}$$

where *n* represents the number of classes, $t_k$ is the truth label, while $P_k$ is the SoftMax probability of *k*th class.

## Experiments

This section first discusses the standard dataset and the pre-processing steps. Secondly, the training methods of the LSTM and temporal transformer architectures are discussed. Lastly, different evaluation parameters are presented, as used in our experiments.

### Dataset

In this work, the dataset BCI III IVa^40^ is used for the recognition of MI signals. The MI-EEG data was recorded by a 128-channel electrode cap by the international 10/20-system, out of which 118 channels are considered. The dataset comprises five subjects labeled as “aa”, “al”, “av”, “aw”, and “ay”. A visual cue was shown for 3.5 s and a total of 280 trials were conducted on all the subjects. Two MI tasks of right hand movement and right foot movement were recorded. The sampling frequency was 1000 Hz. The signals were passed through a band-pass filter between 0.05 and 200 Hz. The most widely adopted dataset for classifying multiclass MI is BCI IV2a^41^, which includes 22 EEG channels and 3 EOG channels. This dataset considered the brain activity of 9 people, while they imagined 4 distinct tasks: left hand, right hand, feet, and tongue movements. A total of 288 trials were conducted on all the subjects. For each task, a total of 196,500 samples were taken from the data gathered from 9 individuals. The sampling frequency for this dataset was 250 Hz and visual cue is shown for 4 s. In addition to a 50 Hz notch filter to reduce the power-line noise, the signals were band-pass filtered between 0.05 and 100 Hz.

In our study, the data is divided into two subsets: a training set and a testing set. The training set consists of 80% of the total data, while the remaining 20% is allocated for testing purposes. To ensure comprehensive analysis, data from all the electrodes are utilized in our experimentation. For the binary class dataset pertaining to the subject “aa”, a total of 58,800 samples are available. Among these, 47,040 samples are randomly used for training, while the remaining 11,760 samples are reserved for testing. For subjects “al” and “av”, 15,680 and 5880 samples are used for testing out of 78,400 and 29,400 samples, respectively. Further, 3920 and 1960 samples are used for testing for subjects “aw” and “ay”, respectively. After combining all the hand and foot data, a total of 196,000 samples are obtained. Out of the obtained samples, 156,800 are randomly used for training and 39,200 are used for testing. This 80:20 split is maintained for all subjects across both classes. A similar process is also utilized for multiclass datasets. In the case of the multi-class dataset concerning the LH dataset for the subject “A01,” a total of 108,640 samples are available. Out of this total, 86,912 samples (approximately 80%) are randomly allocated for training, while the remaining 21,728 samples (approximately 20%) are designated for testing. It is important to note that this ratio is consistently applied to all subjects and classes. Considering the total samples across all subjects, there is a minimum of 982,738 samples in each class. Hence, for considering a uniform balanced dataset, we consider 982,500 samples in each class. Consequently, we have a cumulative total of 196,500 test samples (20% of 982,500) across individual classes.

### Training parameters

Both the models are trained on RTX 2080Ti GPU having 12 GB of VRAM. The learning rate is set to $lr=0.0001$, and the batch size is taken as 200. Sparse categorical cross entropy is used as a loss function. Adaptive moment estimation (ADAM) optimizer is used for training the models. The LSTM model is trained for 30 epochs to achieve reasonable model convergence. Table 1 shows the parameters used by the transformer model for binary classification. In the binary class dataset, an 80:20 partitioning ratio is maintained for all subjects across both classes. It is worth mentioning that the total input size in this scenario becomes 196,000 $\times$ 118, where 118 represents the total number of EEG channels.

**Table 1:** Parameters for proposed transformer-based MI EEG Recognition model.

Model setting		Training setting	
Number of transformers	4	Input shape	N $\times$ 118 $\times$ 1
Dimension of token	118 $\times$ 1	Batch size	200
Number of attention heads	4	Learning rate	0.0001
		Training epochs	278
Dataset size 196,000 $\times$ 118 $\times$ 1			
Total trainable parameters 44,106			
Training time epoch 44,106			

### Validation parameters

Accuracy, sensitivity, specificity, precision, and F1-score are used as performance measures to validate the suggested approach. These measures are obtained as follows:

6 $$\begin{aligned} Accuracy= & {} \frac{TP+TN}{TP+TN+FP+FN}\times 100, \end{aligned}$$

7 $$\begin{aligned} Sensitivity= & {} \frac{TP}{TP+FN}\times 100, \end{aligned}$$

8 $$\begin{aligned} Specificity= & {} \frac{TN}{TN+FP}\times 100, \end{aligned}$$

9 $$\begin{aligned} Precision= & {} \frac{TP}{TP+FP}\times 100, \end{aligned}$$

10 $$\begin{aligned} F1-score= & {} \frac{2\times TP}{FN+FP+2\times TP}\times 100, \end{aligned}$$

where a true positive (TP) precisely determines the presence of a condition or trait. A true negative (TN) establishes the absence of a condition or trait. A false positive (FP) wrongly concludes that a condition or trait is present, while a false negative (FN) incorrectly identifies the absence of a condition or trait.

## Results and discussion

In this section, results for binary class and multi-class scenarios are discussed for both the architectures. We first consider the binary classification problem of BCI III IVa dataset and then multiclass classification problem of BCI competition IV 2a dataset .

**Table 2:** Performance evaluation of LSTM and transformer models for binary class dataset.

Model	Evaluation parameters				
	Accuracy (%)	Senstivity (%)	Specificity (%)	Precision (%)	F-1 Score (%)
LSTM	85.2	82.5	88.4	89.6	85.9
Transformer	99.7	99.7	99.7	99.7	99

### Binary class

**Figure 6:**
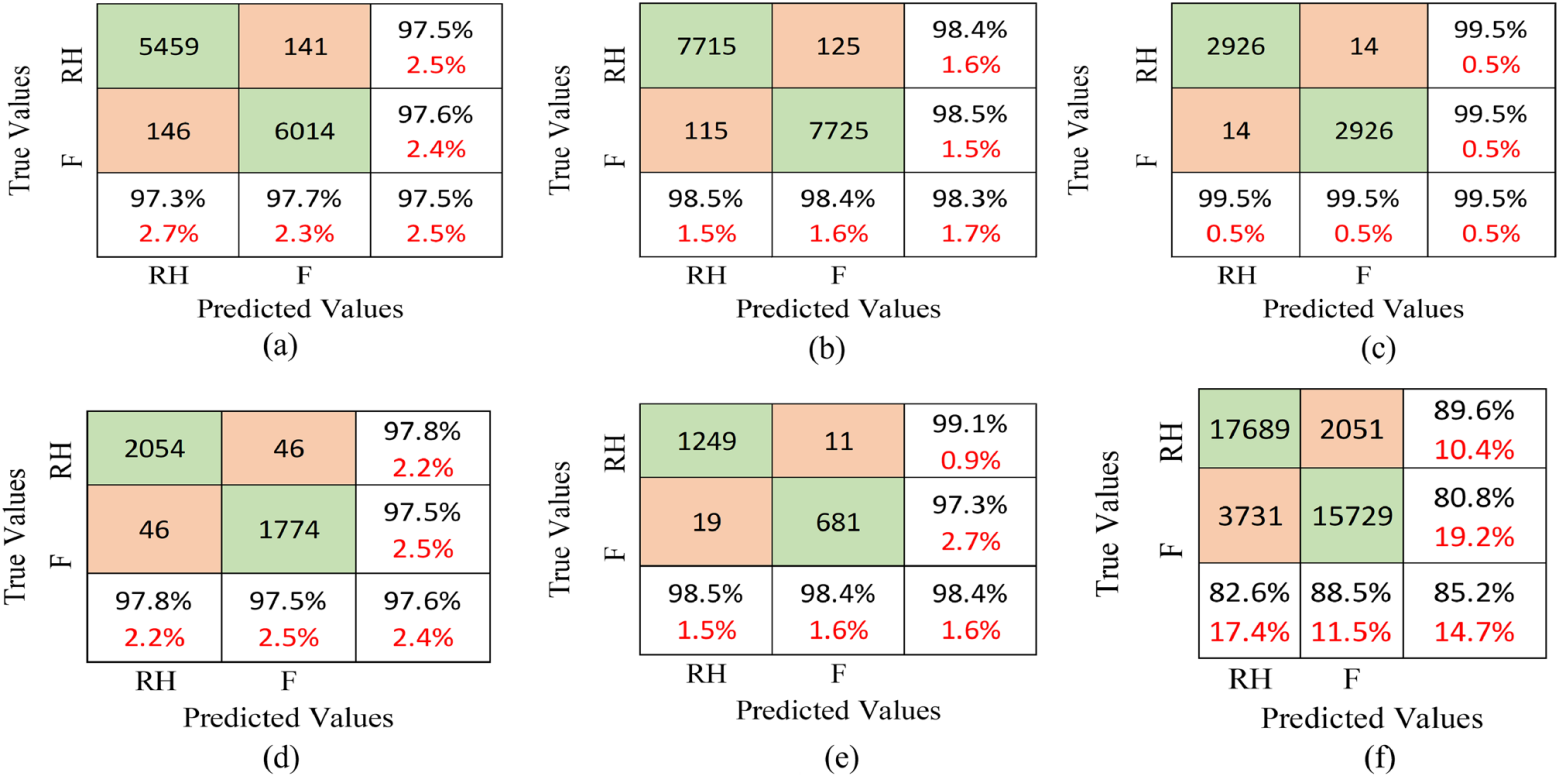
Confusion matrices for LSTM-based MI EEG recognition model for the subjects (**a**) “aa”, (**b**) “al”, (**c**) “av”, (**d**) “aw”, (**e**) “ay”, and (**f**) All subjects.

Foot and right hand are the two classes that need to be recognized in this case. Firstly, results using LSTM architecture are presented. The motion recognition of foot and right hand is analyzed for the individual subjects and the confusion matrices for all five subjects “aa”, “al”, “av”, “aw”, and “ay” are depicted in Fig. 6a–e, respectively. In Fig. 6a, proposed methodology attained classification accuracy of 97.5% by correctly identifying 6014 samples of foot. On the contrary, 146 samples of foot are identified incorrectly for the subject “aa”. For right hand, the number of correctly identified samples are 5459, while 141 samples are not identified correctly. The classification accuracy of 98.3% is achieved for subject “av”, as shown in Fig. 6b. Here, 7725 of the 7850 foot samples are successfully recognised as foot, while 125 are mistakenly identified. Out of the 7830 right hand samples, 7715 are correctly recognised as right hand, while 115 are incorrect. For subject “al”, classification accuracy of 99.5% is achieved by correctly identifying 2926 samples and 14 are wrongly identified for both the classes as illustrated in Fig. 6c. According to Fig. 6d, the classification accuracy for subject “aw” is 97.6%. Correctly identified foot and right hand samples are 1774 and 2054, respectively, and 46 are wrongly identified for both the classes. In Fig. 6e, 681 foot samples are correctly identified and 11 are identified incorrectly. For right hand, the number of correctly identified samples are 1249, while 19 samples are wrongly identified. The classification accuracy achieved by suggested approach is 98.4% for subject “ay”. Figure 6f illustrates the combined confusion matrix for all subjects’ foot and right hand, where the foot and hand data of all the subjects is combined. For the binary class dataset for the subject “aa”, 22,400 samples of the foot are used for training and 5600 are used for testing out of a total of 28,000 samples. Similarly, data for all the subjects is divided into 80:20 ratio for training and testing. Combining the data for all subjects, we obtained a total of 19,460 test samples for foot and 19,740 test samples for hand. The overall classification accuracy for the binary data is 85.2%. Table 2 shows the evaluation parameters for LSTM and transformer for binary class dataset.

**Figure 7:**
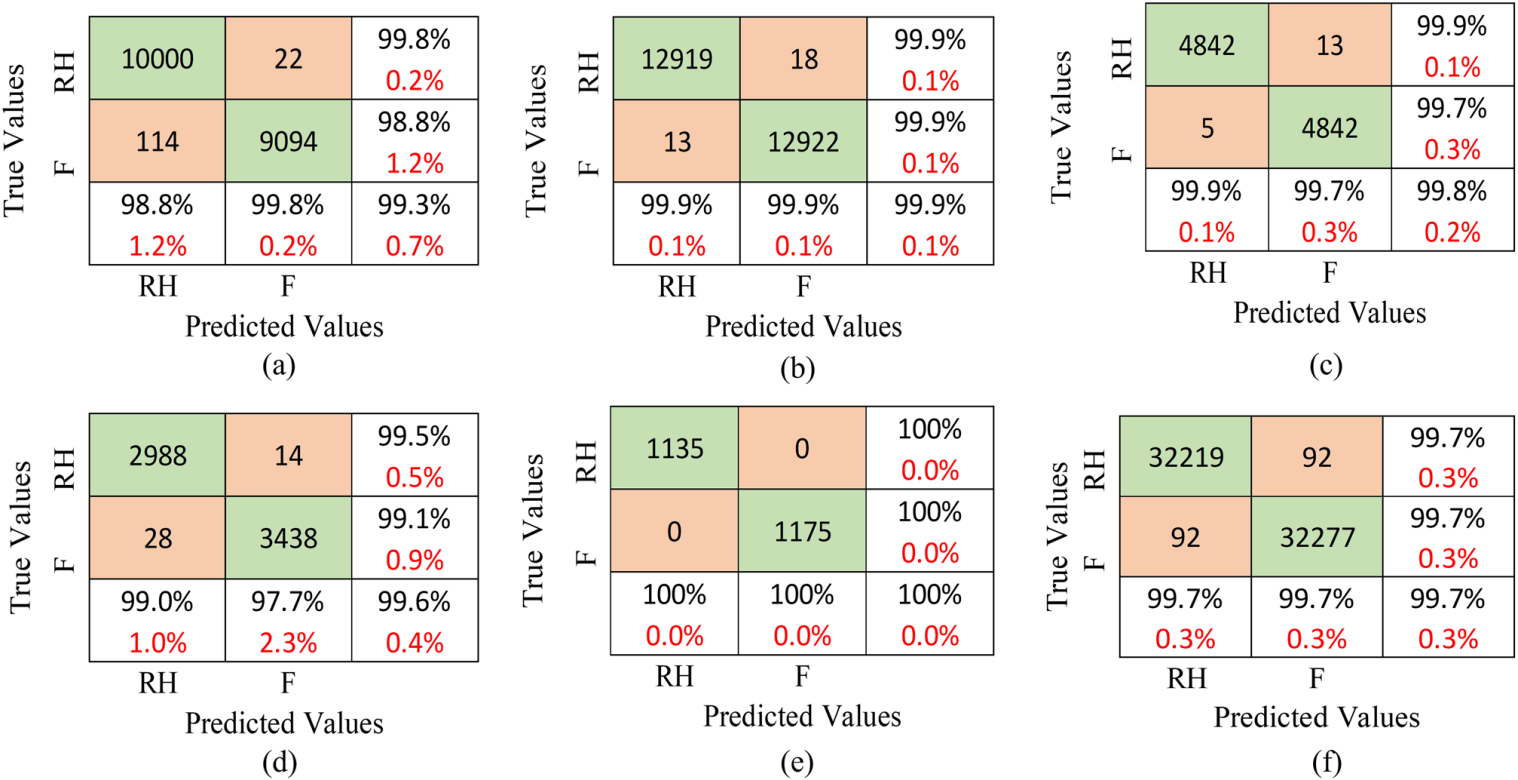
Confusion matrices for the transformer-based MI EEG recognition model for the subjects (**a**) “aa”, (**b**) “al”, (**c**) “av”, (**d**) “aw”, (**e**) “ay”, and (**f**) all subjects.

**Figure 8:**
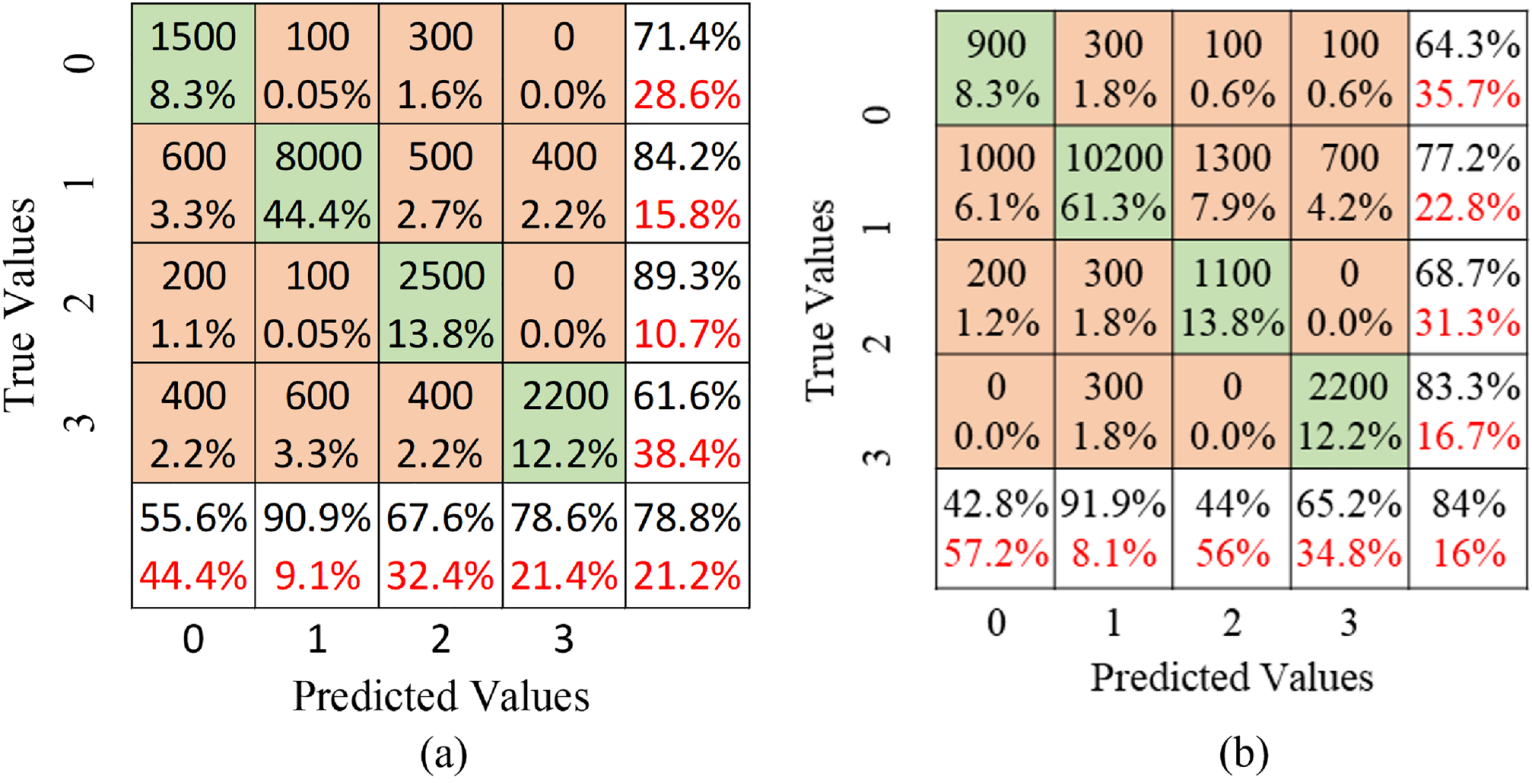
Confusion matrices for multiple classes obtained by (**a**) LSTM-based MI EEG recognition model, and (**b**) Transformer-based MI EEG recognition model.

**Table 3:** Performance evaluation of LSTM and transformer models for multi-class dataset.

Model	Class	Evaluation parameters				
		Accuracy (%)	Senstivity (%)	Specificity (%)	Precision (%)	F-1 score (%)
LSTM	Class-0	90	71.4	99.2	55.6	62.5
	Class-1	87.2	84.2	90.5	90.9	87.4
	Class-2	90	71.4	99.2	55.6	62.5
	Class-3	88.9	61.1	95.8	78.6	68.8
Transformer	Class-0	90.5	64.2	92.7	29	39.9
	Class-1	78.3	83.6	81.3	91.8	87.5
	Class-2	89.4	68.8	92	45.8	54.5
	Class-3	93.9	83.3	95.1	65.2	73.2

Next, we present the results for transformer architecture. In Fig. 7a–e, confusion matrices for all five subjects are shown, whereas Fig. 7f depicts confusion matrix for the combined data. The proposed methodology yields a classification accuracy of 99.3% for subject “aa”. 9094 samples of the foot are identified correctly while 22 samples are identified incorrectly. For the right hand, 10,000 samples are identified correctly and 114 samples are wrongly identified. The classification accuracy of 99.9% is achieved for subject “al” wherein 12,922 out of the 12,940 foot samples are recognised successfully but 18 samples are identified incorrectly. 12,919 out of the 12,932 right hand samples are correctly recognised. Classification accuracy of 99.8% is obtained for subject “av” by correctly identifying 4842 samples for both the classes. For subject “aw”, 99.6% accuracy is achieved. 3438 foot and 2988 right hand samples are classified correctly. Lastly, for subject “ay”, all the samples are correctly identified for both the classes with an accuracy of 100%. In Fig. 7f, the correctly identified samples are 32,277 and 32,219 for foot and right hand, respectively, while 92 samples for both classes are wrongly identified. The classification accuracy achieved by the suggested approach is 99.7% for the combined data.

The performance metrics, such as accuracy, sensitivity, specificity, precision and F-1 scores, are evaluated and shown in Table 2. For LSTM, we achieve 85.2%, 82.5%, 88.4%, 89.6%, and 85.9% values for accuracy, sensitivity, specificity, precision, and F-1 score, respectively. Similarly, for transformer, these performance measures are 99.7%, 99.7%, 99.7%, 99.7%, and 99%, respectively.

**Table 4:** Comparison of classification accuracy of proposed methodology with existing techniques.

Sr. no.	Author	Methodology	Dataset	Accuracy (%)
1	Dai et al.^13^	Transfer kernel CSP	BCI III IVa	91.2
2	Taheri et al.^18^	CNN/ReLU	BCI III IVa	96.34
3	Song et al.^20^	Transformer	BCI IV 2a	84.2
			BCI IV 2b	82.59
4	Yongkoo et al.^42^	CSP feature	BCI III IVa	84.4
5	Ma et al.^43^	CNN-transformer	BCI IV 2a	83.9
6	Zhang et al.^44^	CNN/LSTM	BCI IV 2a	83
7	Proposed methodology		BCI III IVa	99.5
			BCI IV 2a	84

### Multi-class

The four classes that need to be identified in the BCI competition IVa multi-class dataset are left hand, right hand, foot, and tongue. Multiply each element of the confusion matrix by 40 to get the sample value. Firstly, the outcomes of the LSTM architecture are shown. The left hand is labelled as class 0, right hand as class 1, feet as class 2, and tongue as class 3, as shown in Fig. 8. The confusion matrix for LSTM is depicted in Fig. 8a. The proposed methodology attained classification accuracy of 78.8% by correctly identifying 1500 samples of left hand, 8000 samples of right hand, 2500 samples of feet, and 2200 samples of tongue. On the other hand, 600, 200, and 400 samples of classes 1, 2, and 3, respectively, are wrongly classified as class 0. Further, a total of 800, 1200, and 400 samples are identified incorrectly as classes 1, 2, and 3, respectively.

Next, the results are shown for the transformer architecture. Figure 8b shows that 900, 10,200, 1100 and 2200 samples of classes 0, 1, 2, and 3, respectively, are correctly classified. On the other hand, a total of 1200, 900, 1400, and 800 samples are identified incorrectly as classes 0, 1, 2, and 3, respectively. The performance metrics, including accuracy, sensitivity, specificity, precision, and F-1 score, are assessed and shown in Table 3. Transformer produces better accuracy than LSTM for classes 0 and 3, while LSTM provides better accuracy for classes 1 and 2. In terms of F1-score, transformer provides better score for classes 1 and 3, while LSTM outperforms for the other two classes.

The proposed approach processes the dataset of BCI competition III IVa and BCI IV 2a in this paper. Here, the signal is passed through a transformer encoder where seven layers of convolution layers are used. Multi-head attention decreases the system’s computational time as it supports parallel computing of data. Due to multi-head attention, it provides better accuracy. Residual is always forwarded to the next layer which also helps in improving the accuracy of the system. The proposed methodology attained an accuracy of 99.7% and 84% whereas the associated loss is 0.0098% and 16% for binary class and multi-class datasets, respectively. Our results are on par with state-of-the-art methods that employ deep learning techniques on MI recognition tasks by converting the 1-dimensional (1D) data into 2-dimensional (2D) images. Our proposed method offers the advantage of reduced computational complexity by directly applying LSTM/transformer models to the raw EEG data (1D data). Table 4 compares the performance of the proposed methodology along with other methodologies. From this table, we can observe that the proposed methodology has the highest classification accuracy of 99.7% for binary class dataset as compared to other methods. The classification accuracy of other algorithms lies between 84.4 and 96.34%, which is lower than the proposed algorithm. Yongkoo et al.^42^ achieved high accuracy for the “aa” subject, however, accuracy is lower for all other subjects.

For the multiclass datset, Song et al.^20^ considered a transformer-based method and attained accuracy of 84.2%. But, the author has performed preprocessing on the dataset along with the spatial filtering and temporal input to the transformer encoder, leading to additional computational complexity. Instead of using the raw 1D data, they applied spatial filtering and transformed the input data of the transformer model in both spatial and temporal domains. CNN-transformer is used by Ma et al.^43^ and accuracy of 83.9% is achieved. In another work^44^, authors used CNN-LSTM architecture to achieve 83% accuracy.

## Conclusion

In this paper, a robust and efficient method for MI-EEG signal classification has been proposed. Two different architectures, LSTM and transformer, are considered and their performance is compared for the recognition of MI signals. It has been established that the proposed method outperforms existing state-of-the-art approaches on BCI competition III IVa dataset. Further, we have experimentally shown that transformers perform exceptionally well over LSTM models in encoding long-term dependencies in time sequence data. The transformer-based network is faster and easily multi-processed for faster performance. It achieved high accuracy and efficiency, thus, can be utilized in real-time applications. In future, the performance of the model could be evaluated on some other datasets for MI-EEG. Further, the proposed method could be explored for various other applications, such as detection of brain diseases. For the multi-class problem, the classification accuracy got limited to 84% because of the high correlation amongst the different classes. Some variants of the model could be investigated to address this issue.

## Data Availability

The datasets generated and/or analysed during the current study are available at https://www.bbci.de/competition/iii/desc_IVa.html and https://www.bbci.de/competition/iv/#dataset1.
